# Choice of Estimated GFR Concept and Implications for Cystatin C to Creatinine Ratio Among Hospitalized Older Adults

**DOI:** 10.1016/j.ekir.2024.04.059

**Published:** 2024-05-04

**Authors:** Esben Iversen, Louise Westberg Strejby Christensen, Aino Leegaard Andersen, Rikke Lundsgaard Nielsen, Morten Damgaard, Trine Meldgaard Lund, Mads Hornum, Ove Andersen, Morten Baltzer Houlind

**Affiliations:** 1 Department of Clinical Research, Copenhagen University Hospital Amager and Hvidovre, Hvidovre, Denmark; 2 The Capital Region Pharmacy, Herlev, Denmark; 3 Department of Clinical Medicine, University of Copenhagen, Copenhagen, Denmark; 4 Department of Clinical Physiology and Nuclear Medicine, Center for Functional and Diagnostic Imaging and Research, Copenhagen University Hospital Amager and Hvidovre, Hvidovre, Denmark; 5 Department of Drug Design and Pharmacology, University of Copenhagen, Copenhagen, Denmark; 6 Department of Nephrology, Copenhagen University Hospital Rigshospitalet, Copenhagen, Denmark; 7 Emergency Department, Copenhagen University Hospital Amager and Hvidovre, Hvidovre, Denmark

**Keywords:** creatinine, cystatin C, estimated glomerular filtration rate (eGFR), hospitalized older adults, nonrenal factors, selective glomerular hypofiltration

## Introduction

Older adults (age ≥65 years) account for nearly half of all hospitalizations in both Europe and the United States.^1^ Hospitalized older adults are uniquely vulnerable to inaccurate creatinine-based estimates of glomerular filtration rate (eGFRcre) due to nonrenal factors such as sarcopenia^2^ and malnutrition^3^ that affect creatinine levels. Cystatin C-based estimates (eGFRcys) often yield large and clinically relevant discrepancies from eGFRcre^4^, but the cause of these discrepancies is unclear. One possible explanation is that cystatin C is influenced by a unique set of nonrenal factors, such as inflammation,^5^ that cause eGFRcys to diverge from eGFRcre. An alternative explanation is that changes to the renal filtration barrier cause a relative decrease in cystatin C filtration without affecting creatinine filtration, a phenomenon referred to as selective glomerular hypofiltration.^6^

Low eGFRcys-to-eGFRcre ratio is associated with adverse clinical outcomes ranging from cardiovascular disease to mortality.^7^^,^^8^ It has also been proposed as an indication for direct kidney function measurement^9^ and as a screening test for sarcopenia or malnutrition.S1 However, there is no consensus on what cutoff should be used to define a “low” ratio or which eGFR concept should be used to calculate this ratio. Most clinical laboratories in Europe and the United States use equations from the Chronic Kidney Disease Epidemiology Collaboration (CKD-EPI). However, Europe continues to use the original CKD-EPI concept (2009 CKD-EPIcre and 2012 CKD-EPIcys) based on recommendations from the European Renal Association,S2 whereas the United States is transitioning to the revised CKD-EPI concept (2021 CKD-EPIcre and 2023 CKD-EPIcys) based on recommendations from the National Kidney Foundation and American Society of Nephrology.S3 Meanwhile, the European Kidney Function Consortium (EKFC) developed its own set of equations (EKFCcre and EKFCcys) that may be more appropriate than CKD-EPI in European settings.S4

We have previously shown how switching between these eGFR concepts impacts clinical decisions for hospitalized older adultsS5 and other high-risk patient groups.S6 Therefore, we hypothesized that switching between eGFR concepts would also affect eGFRcys-to-eGFRcre ratio, which could impact clinical care if this metric becomes a standard diagnostic and/or prognostic tool.

## Results

The original OptiNAM trial enrolled 193 patients, and the final study population for this analysis included 106 patients (median age: 78.3 years, median measured GFR [mGFR]: 62.9 ml/min per 1.73 m^2^, and 58% female) (Table 1). Patients with an eGFRcys-to-eGFRcre ratio <0.7 had lower median mGFR, higher rates of malnutrition and smoking, and higher levels of acute and chronic inflammation as reflected by median C-reactive protein and soluble urokinase plasminogen activator receptor, respectively. Performance metrics for each eGFR equation relative to mGFR are given in Supplementary Table S4.

**Table 1:** Patient characteristics by eGFRcys-to-eGFRcre ratio according to each eGFR concept

Characteristics	Overall	eGFRcys-to-eGFRcre ratio according to:					
		Original CKD-EPI		Revised CKD-EPI		EKFC	
		≥0.7	<0.7	≥0.7	<0.7	≥0.7	<0.7
Number	106 (100%)	70 (66%)	36 (34%)	54 (51%)	52 (49%)	96 (91%)	10 (9%)
Sex (female)	61 (58%)	39 (56%)	22 (61%)	34 (63%)	27 (52%)	59 (62%)	2 (20%)
Age (yr)	78 (74-84)	78 (74–83)	79 (75–87)	78 (74–82)	80 (75–87)	78 (74–83)	77 (71–85)
mGFR^a^ (ml/min per 1.73 m^2^)	63 (47–76)	69 (51–78)	52 (43–65)	69 (52–78)	56 (44–70)	64 (48–76)	55 (44–72)
Body mass index (kg/m^2^)	26 (22–31)	26 (23–31)	26 (21–31)	26 (22–30)	26 (22–31)	26 (22–30)	30 (22–34)
Malnutrition^b^	68 (64%)	40 (57%)	28 (78%)	30 (56%)	38 (73%)	60 (63%)	8 (80%)
Smoking^c^	77 (73%)	50 (71%)	27 (75%)	36 (67%)	41 (79%)	69 (72%)	8 (80%)
Hypertension	52 (49%)	35 (50%)	17 (47%)	29 (54%)	23 (44%)	49 (51%)	3 (30%)
Diabetes mellitus	20 (19%)	11 (16%)	9 (25%)	7 (13%)	13 (25%)	17 (18%)	3 (30%)
C-reactive protein (mg/l)	4.5 (1.9–9.1)	3.7 (1.7–8.3)	6.6 (4.2–10.6)	3.3 (1.3–6.3)	6.6 (3.7–14.0)	4.5 (1.9–10.2)	4.9 (3.9–8.0)
suPAR (ng/ml)^d^	4.6 (3.6–5.9)	4.2 (3.3–5.2)	5.5 (4.5–6.6)	3.9 (3.2–5.0)	5.4 (4.3–6.6)	4.6 (3.5–5.9)	4.7 (4.6–5.6)

CKD-EPI, Chronic Kidney Disease Epidemiology Collaboration; cre, creatinine; cys, cystatin C; eGFR, estimated glomerular filtration rate; EKFC, European Kidney Function Consortium; IQR, interquartile range (Q1–Q3); mGFR, measured glomerular filtration rate; suPAR, soluble urokinase plasminogen activator receptor.

All values are number (%) or median (IQR).

a mGFR was determined by single-injection plasma clearance of 99mTechnetium-diethylenetriaminepentaacetic acid (99mTc-DTPA).

b Malnutrition was defined as a score of ≤11 on the Mini Nutritional Assessment Short-Form.S8

c Smoking includes both current and former smokers.

d suPAR is a biomarker of systemic chronic inflammation.S9

For all 3 eGFR concepts, median eGFR was lower for eGFRcys compared to eGFRcre (Table 2). Median eGFRcys-to-eGFRcre ratio was lowest (furthest from 1) for the revised CKD-EPI concept and highest (closest to 1) for the EKFC concept. An eGFRcys-to-eGFRcre ratio <0.7 occurred in 34.0%, 49.1%, or 9.4% of patients according to the original CKD-EPI, revised CKD-EPI, or EKFC concept, respectively. No patients in the cohort had an eGFRcre-to-eGFRcys ratio <0.7 according to any eGFR concept. A similar trend was observed when using cutoffs of <0.6 or <0.8, or when defining “eGFR discrepancy” as an absolute difference of >15 ml/min per 1.73 m^2^ (Supplementary Table S5).

**Table 2:** eGFRcys-to-eGFRcre ratio among 106 hospitalized older adults according to each eGFR concept

eGFR concept and equations	Median (IQR) eGFR in ml/min per 1.73 m^2^	Median (IQR) eGFRcys-to-eGFRcre ratio	Number (%) of patients with eGFRcys-to-eGFRcre ratio:		
			<0.6	<0.7	<0.8
Original CKD-EPI concept					
2009 CKD-EPIcre	65.4 (53.4–81.4)	0.77 (0.64–0.89)	16 (15.1%)	36 (34.0%)	61 (57.5%)
2012 CKD-EPIcys	48.1 (36.7–62.6)				
Revised CKD-EPI concept					
2021 CKD-EPIcre	71.0 (58.0–87.5)	0.70 (0.62–0.84)	24 (22.6%)	52 (49.1%)	71 (67.0%)
2023 CKD-EPIcys	48.1 (36.8–63.3)				
EKFC concept					
EKFCcre	59.3 (47.2–72.0)	0.89 (0.79–1.0)	5 (4.7%)	10 (9.4%)	31 (29.2%)
EKFCcys	50.6 (39.9–63.9)				

CKD-EPI, Chronic Kidney Disease Epidemiology Collaboration; cre, creatinine; cys, cystatin C; eGFR, estimated glomerular filtration rate; EKFC, European Kidney Function Consortium; IQR, interquartile range; mGFR, measured glomerular filtration rate.

## Discussion

Regardless of eGFR concept, median eGFR was lower when calculated using cystatin C versus creatinine. This finding can be explained at least in part by low protein intake (64% of patients had malnutrition) affecting creatinine concentration, and high inflammation (median C-reactive protein level was 4.5 mg/l, median soluble urokinase plasminogen activator receptor level was 4.6 ng/ml) affecting cystatin C concentration. Alternatively, this finding could be due to selective hypofiltration of cystatin C among hospitalized older adults. In either case, our results demonstrate that choice of eGFR concept has a substantial impact on how many patients would be classified as having a “low” eGFRcys-to-eGFRcre ratio using a cutoff of <0.7: fewer than 10% of patients for the EKFC concept versus nearly 50% of patients for the revised CKD-EPI concept. The relatively small discrepancies between EKFCcre and EKFCcys likely reflect the construction of these equations, which share the same mathematical structure and differ only in a single value (the Q-factor).

It is typical to comment on the performance of eGFR equations relative to mGFR. Among the equations used in this study, the 2009 CKD-EPIcre and EKFCcre equations performed best (smallest bias and highest accuracy), whereas EKFCcys performed best among cystatin C-based equations. However, we have previously shown that equations combining creatinine and cystatin C (eGFRcre-cys) outperform both eGFRcre and eGFRcys in this cohort.S5 Furthermore, it is important to consider whether the exogenous filtration markers used to determine mGFR accurately reflect the filtration of both creatinine and cystatin C in the first place. Traditional mGFR markers (including 99mTc-DTPA) are similar in size to creatinine (0.1 kDa), so they will not detect changes to the renal filtration barrier that do not affect creatinine filtration. The cystatin C molecule is much larger (13.3 kDa), so it is plausible that changes to cystatin C filtration are not detected by traditional mGFR markers. Therefore, the true cause of discrepancies between eGFRcys and eGFRcre (nonrenal factors or selective hypofiltration) must be clarified by comparing traditional mGFR markers with mGFR markers similar in size to cystatin C. Earlier studies have demonstrated selective hypofiltration using neutral dextrans (>5 kDa)S10,S11, but this finding has not been replicated in high-risk populations with an outsized effect of nonrenal factors, such as hospitalized older adults.

We did not assess clinical outcomes as part of this study. Earlier studies have consistently found that large discrepancies between eGFRcys and eGFRcre are associated with adverse clinical outcomes. A significant limitation of these studies is that there is no consensus on how they define “large discrepancies.” As we show here, switching between eGFR concepts alone can drastically alter how risk groups are identified for the purpose of investigating clinical outcomes. Given the potential for eGFR discrepancies to be used for diagnostic and/or prognostic purposes, it is essential that clinicians and researchers agree on how these discrepancies are defined (i.e., which eGFR concept should be used). The specific choice of cutoff for eGFRcys-to-eGFRcre ratio (e.g., 0.6, 0.7, or 0.8) will depend on the medical community’s desired sensitivity and specificity for specific clinical outcomes. These cutoffs should also be developed and tested in large international patient cohorts across diverse clinical settings.

In summary, researchers investigating clinical outcomes based on discrepancies between eGFRcys and eGFRcre must be aware that choice of eGFR concept will influence the proportion of patients with a “large” discrepancy and, therefore, any observed association with clinical endpoints. Similarly, renal societies in Europe and the United States should consider how their recommendations for choice of eGFR concept will affect the identification of eGFR discrepancies in practice. We found that the revised CKD-EPI concept yields the largest discrepancies between eGFRcre and eGFRcys for hospitalized older adults, making the selection of filtration marker highly consequential for individual patients. These discrepancies were much smaller for the original CKD-EPI and EKFC concepts, reflecting consistent eGFR output regardless of which filtration marker is used. The cause of eGFR discrepancies, and therefore the most appropriate eGFR concept, should be clarified by comparing the filtration of exogenous filtration markers that differ in molecular size.

## Disclosure

OA is a named inventor on patents covering soluble urokinase plasminogen activator receptor owned by Copenhagen University Hospital Amager & Hvidovre, Hvidovre, Denmark and licensed to ViroGates A/S. All the other authors declared no competing interests.

## Supplementary Material

Supplementary File (PDF) **Supplementary Methods. Table S1.** Study eligibility criteria for the original OptiNAM trial and subsequent analysis. **Table S2.** Assays and instruments used to measure biomarkers in plasma. **Table S3.** List of equations used to estimate glomerular filtration rate. **Table S4.** Performance of each eGFR equation relative to mGFR. **Table S5.** Number (%) of patients with absolute difference of >15 ml/min per 1.73 m^2^ between eGFRcys and eGFRcre according to each eGFR concept. **STROBE Checklist.**

## Footnotes

Supplementary File (PDF)

**Supplementary Methods.**

**Supplementary Tables.**

**Supplementary References.**

**STROBE Checklist.**
