# Supplementary material for: Choice of Estimated GFR Concept and Implications for Cystatin C to Creatinine Ratio Among Hospitalized Older Adults
Source: Kidney Int Rep. 2024 May 4;9(7):2295–8. doi: 10.1016/j.ekir.2024.04.059 (PMC11284434; doi:10.1016/j.ekir.2024.04.059)
Supplement: Supplementary File (PDF) — Supplementary Methods. Table S1. Study eligibility criteria for the original OptiNAM trial and subsequent analysis. Table S2. Assays and instruments used to measure biomarkers in plasma. Table S3. List of equations used to estimate glomerular filtration rate. Table S4. Performance of each eGFR equation relative to mGFR. Table S5. Number (%) of patients with absolute difference of >15 ml/min per 1.73 m2 between eGFRcys and eGFRcre according to each eGFR concept. STROBE Checklist. [file mmc1.pdf]

Supplementary Material

Choice of Estimated GFR Concept and Implications for Cystatin C to Creatinine Ratio Among Hospitalized Older Adults

E. Iversen*, L.W.S. Christensen, A.L. Andersen, R.L. Nielsen, M. Damgaard, T.M. Lund, M. Hornum, O. Andersen, M.B. Houlind

*Correspondence: Esben Iversen (esben.iversen@regionh.dk)

Supplementary Methods
Supplementary Tables
Supplementary References
STROBE Checklist

Supplementary Methods
The methods used in this article are largely identical to those described in an earlier publication (doi: 10.1053/j.ajkd.2023.05.004). Relevant information has been reproduced below.

The data used in this article was obtained from a randomized controlled trial investigating the effectiveness of an intervention to optimize nutrition and medication (OptiNAM) among older patients in the emergency department (ED) at risk of malnutrition. The OptiNAM trial was approved by Denmark's Ethics Committee for the Capital Region (identifier: H-18023853) and Data Protection Agency (identifier: VD-2018-390) and registered at www.clinicaltrials.gov (identifier: NCT03741283). The study was conducted in accordance with the Declaration of Helsinki, and all study participants provided written and oral informed consent. A study protocol for the OptiNAM trial has previously been published by Andersen et al. (doi: 10.1186/s13063-021-05456-6).

The OptiNAM trial included Caucasian adults age ≥65 years presenting to the Emergency Department of Copenhagen University Hospital Amager and Hvidovre, Hvidovre, Denmark (hereafter: Hvidovre Hospital) between October 2018 and April 2021. All participants were recruited within 36 hours of hospitalization. A full list of exclusion criteria for the original study and subsequent analysis are given in **Table S1**.

All GFR measurements were performed on Wednesday mornings (typically between 9am and 12pm) at the Department of Clinical Physiological and Nuclear Medicine, Centre for Functional and Diagnostic Imaging and Research, Hvidovre Hospital. If possible, GFR measurement was performed during hospitalization (after study recruitment). Otherwise, study participants were asked to return for GFR measurement as soon as possible after discharge. Measured GFR (mGFR) was determined by single-injection plasma clearance of 99mTechnetium–diethylenetriaminepentaacetic acid (99mTc-DTPA) using standard methods36,37. In short, 40 MBq of 99mTc-DTPA was injected intravenously, and samples were collected from venous blood at 180, 200, 220, and 240 min after injection for patients with eGFR >30 mL/min, or 180, 210, 240, 270, and 300 min after injection for patients with eGFR ≤30 mL/min.

Demographic information and physical characteristics including weight and height were recorded on the same day as GFR measurement. Disease status including admission diagnoses and comorbidities was determined by International Classification of Diseases (ICD-10) diagnosis codes in electronic patient records. Blood samples were collected immediately prior to GFR measurement, stored at –80 °C in a biobank at Hvidovre Hospital, and analyzed using standard methods (**Table S2**).

GFR was estimated using equations from the original CKD-EPI, revised CKD-EPI, and EKFC concepts (**Table S3**). The performance of each eGFR equation compared to mGFR was assessed by bias and P30 accuracy in the study population. Bias was defined as the median value of [eGFR–mGFR], and P30 was defined as the proportion of eGFR values within 30% of mGFR. Confidence intervals were generated by bootstrapping with 10,000 iterations. Statistical analysis was performed in R version 4.3.0 (R Foundation for Statistical Computing, Vienna, Austria).

Supplementary Tables

Table S1. Study eligibility criteria for the original OptiNAM trial and subsequent analysis.

Table S2. Assays and instruments used to measure biomarkers in plasma.

Table S3. List of equations used to estimate glomerular filtration rate.

Table S4. Performance of each eGFR equation relative to mGFR.

Table S5. Number (%) of patients with absolute difference of >15 mL/min/1.73m^2 between eGFRcys and eGFRcre according to each eGFR concept.

Table S1. Study eligibility criteria for the original OptiNAM trial and subsequent analysis.

OptiNAM inclusion criteria	OptiNAM exclusion criteria	Additional exclusion criteria
Caucasian	Admission due to terminal illness or suicide attempt	Declined GFR measurement*
Age ≥65 years	Isolation due to infection precautions	Excluded from GFR measurement due to death, dialysis, or clinical signs of edema or ascites
Presenting to the emergency department	Inability to effectively communicate due to language barrier, hearing impairment, or speech impediment	Prior amputation, use of an immunosuppressant, or acute kidney injury** on the day of GFR measurement
	Inability to cooperate due to cognitive impairment or any other reason	

*Patients could decline participation in the GFR measurement portion of the clinical trial for any reason.

**Acute kidney injury was defined as an increase in serum creatinine of ≥0.3 mg/dL or ≥50% from baseline, where baseline was defined as the lowest creatinine value obtained during hospitalization.

GFR, glomerular filtration rate; OptiNAM, Optimization of Nutrition and Medication

Table S2. Assays and instruments used to measure biomarkers in plasma.

Marker	Assay	Manufacturer	Instrument
Creatinine	Enzymatic, standardized against IDMS	Roche	Cobas 8000 c702
Cystatin C	Immunoturbidimetry	Roche	Cobas 8000 c502
C-reactive protein (CRP)	Particle-enhanced immunoturbidimetry	Roche	Cobas 8000 c702
Soluble urokinase plasminogen activator receptor (suPAR)	Enzyme-linked immunoassay	Virogates A/S	suPARnostic®

Table S3. List of equations used to estimate glomerular filtration rate.

	Sex	Pcre	Pcys	Equation*
**2009 CKD-EPIcre**[S12]				
	Male	≤ 0.9		$141 \times (P_{Cre}/0.9)^{-0.411} \times 0.9929^{Age}$
		> 0.9		$141 \times (P_{Cre}/0.9)^{-1.209} \times 0.9929^{Age}$
	Female	≤ 0.7		$141 \times (P_{Cre}/0.7)^{-0.329} \times 0.9929^{Age} \times 1.018$
		> 0.7		$141 \times (P_{Cre}/0.7)^{-1.209} \times 0.9929^{Age} \times 1.018$
**2012 CKD-EPIcys**[S13]				
	Male		≤ 0.8	$133 \times (P_{Cys}/0.8)^{-0.499} \times 0.9962^{Age}$
			> 0.8	$133 \times (P_{Cys}/0.8)^{-1.328} \times 0.9962^{Age}$
	Female		≤ 0.8	$133 \times (P_{Cys}/0.8)^{-0.499} \times 0.9962^{Age} \times 0.932$
			> 0.8	$133 \times (P_{Cys}/0.8)^{-1.328} \times 0.9962^{Age} \times 0.932$
**2021 CKD-EPIcre**[S14]				
	Male	≤ 0.9		$142 \times (P_{Cre}/0.9)^{-0.302} \times 0.9938^{Age}$
		> 0.9		$142 \times (P_{Cre}/0.9)^{-1.200} \times 0.9938^{Age}$
	Female	≤ 0.7		$142 \times (P_{Cre}/0.7)^{-0.241} \times 0.9938^{Age} \times 1.012$
		> 0.7		$142 \times (P_{Cre}/0.7)^{-1.200} \times 0.9938^{Age} \times 1.012$
**2023 CKD-EPIcys**[S15]				
			≤ 0.8	$129 \times (P_{Cys}/0.8)^{-0.425} \times 0.9962^{Age}$
			> 0.8	$129 \times (P_{Cys}/0.8)^{-1.328} \times 0.9962^{Age}$
**EKFCcre**[S16]				
	Male	< 0.9		$107.3 \times (P_{Cre}/0.9)^{-0.322} \times 0.990^{(Age-40)}$
		≥ 0.9		$107.3 \times (P_{Cre}/0.9)^{-1.132} \times 0.990^{(Age-40)}$
	Female	< 0.7		$107.3 \times (P_{Cre}/0.7)^{-0.322} \times 0.990^{(Age-40)}$
		≥ 0.7		$107.3 \times (P_{Cre}/0.7)^{-1.132} \times 0.990^{(Age-40)}$
**EKFCcys**[S17]				
			< 0.83 + 0.005 × (Age–50)	$107.3 \times (P_{Cys}/[0.83 + 0.005 \times (Age-50)])^{-0.322} \times 0.990^{(Age-40)}$
			≥ 0.83 + 0.005 × (Age–50)	$107.3 \times (P_{Cys}/[0.83 + 0.005 \times (Age-50)])^{-1.132} \times 0.990^{(Age-40)}$

*The equations presented are specific for non-Black Europeans age >50 years. For the full versions, refer to the given references.

Age, patient age in years; CKD-EPI, Chronic Kidney Disease Epidemiology Collaboration; EKFC, European Kidney Function Consortium; Pcre, plasma creatinine in mg/dL; Pcys, plasma cystatin C in mg/L

Table S4. Performance of each eGFR equation relative to mGFR[a]. All 95% CI were generated by bootstrapping with 10,000 iterations.

**eGFR concept and equations**	**Bias[b]** (95% CI)	**P30[c]** (95% CI)	**P20[c]** (95% CI)
Original CKD-EPI concept			
2009 CKD-EPIcre	+2.6 (–1.3 to +4.7)	91.5 (85.8 to 96.2)	81.1 (73.6 to 88.7)
2012 CKD-EPIcys	–10.4 (–12.2 to –9.0)	80.2 (72.6 to 87.7)	50.0 (40.6 to 59.4)
Revised CKD-EPI concept			
2021 CKD-EPIcre	+7.0 (+3.8 to +9.1)	84.9 (77.4 to 91.5)	65.1 (55.7 to 74.5)
2023 CKD-EPIcys	–10.5 (–12.4 to –8.7)	82.1 (74.5 to 88.7)	50.9 (41.5 to 60.4)
EKFC concept			
EKFCcre	–4.3 (–6.7 to –1.3)	92.5 (86.8 to 97.2)	80.2 (72.6 to 87.7)
EKFCcys	–8.3 (–10.5 to –6.9)	89.6 (83.0 to 95.3)	65.1 (56.6 to 73.6)

[a]mGFR was determined by single-injection plasma clearance of 99mTechnetium-diethylenetriaminepentaacetic acid (99mTc-DTPA).
[b]Bias is defined as the median value of [eGFR–mGFR] in mL/min/1.73m^2 (positive values indicate overestimation, and negative values indicate underestimation).
[c]P30 and P20 are defined as the proportion of eGFR values within 30% or 20%, respectively, of mGFR (larger values indicate higher accuracy).
CKD-EPI, Chronic Kidney Disease Epidemiology Collaboration; cre, creatinine; cys, cystatin C; eGFR, estimated glomerular filtration rate; EKFC, European Kidney Function Consortium; IQR, interquartile range; mGFR, measured glomerular filtration rate

Table S5. Number (%) of patients with absolute difference of >15 mL/min/1.73m2 between eGFRcys and eGFRcre according to each eGFR concept.

**eGFR concept**	**Difference** (eGFRcys – eGFRcre) in mL/min/1.73m^2		
	**< –15** (eGFRcre higher)	**–15 to +15** (concordant)	**> +15** (eGFRcys higher)
Original CKD-EPI concept	49 (46.2%)	56 (52.8%)	1 (0.9%)
Revised CKD-EPI concept	66 (62.3%)	39 (36.8%)	1 (0.9%)
EKFC concept	21 (19.8%)	83 (78.3%)	2 (1.9%)

CKD-EPI, Chronic Kidney Disease Epidemiology Collaboration; eGFR, glomerular filtration rate; EKFC, European Kidney Function Consortium

Supplementary References

S1. S. Yoshida, Y. Nakayama, J. Nakayama, N. Chijiiwa, and T. Ogawa, Assessment of sarcopenia and malnutrition using estimated GFR ratio (eGFRcys/eGFR) in hospitalised adult patients. *Clin Nutr ESPEN*, **48**, 2022, 456–463, doi:10.1016/j.clnesp.2021.12.027.

S2. R.T. Gansevoort, H.J. Anders, M. Cozzolino, et al., What should European nephrology do with the new CKD-EPI equation? *Nephrol Dial Transplant*, **38**, 2022, 1–6, doi:10.1093/ndt/gfac254.

S3. C. Delgado C, M. Baweja, D.C. Crews, et al., A unifying approach for GFR estimation: recommendations of the NKF-ASN task force on reassessing the inclusion of race in diagnosing kidney disease. *Am J Kidney Dis*, **79**, 2022, 268–288, doi:10.1053/j.ajkd.2021.08.003.

S4. P. Delanaye, E. Schaeffner, M. Cozzolino, et al., The new, race-free, Chronic Kidney Disease Epidemiology Consortium (CKD-EPI) equation to estimate glomerular filtration rate: is it applicable in Europe? A position statement by the European Federation of Clinical Chemistry and Laboratory Medicine (EFLM). *Clin Chem Lab Med*, **61**, 2022, 44–47, doi:10.1515/cclm-2022-0928.

S5. E. Iversen, A.K. Bengaard, A.L. Andersen, et al., Performance of panel-estimated GFR among hospitalized older adults. *Am J Kidney Dis*, **82**, 2023, 715–724, doi:10.1053/j.ajkd.2023.05.004.

S6. M.B. Houlind, E. Iversen, V.R. Curovic, et al., Performance of the 2009 CKDEPI, 2021 CKDEPI, and EKFC equations among high-risk patients in Denmark. *Clin Chem Lab Med*, **61**, 2023, e192–e195, doi:10.1515/cclm-2023-0041.

S7. A.L. Andersen, M.B. Houlind, R.L. Nielsen, et al., Optimization of Nutrition and Medication (OptiNAM) for acutely admitted older patients: protocol for a randomized single-blinded controlled trial. *Trials*, **22**, 2021, 616, doi:10.1186/s13063-021-05456-6.

S8. L.Z. Rubenstein, J.O. Harker, A. Salvà, Y. Guigoz, and B. Vellas, Screening for undernutrition in geriatric practice: developing the short-form mini-nutritional assessment (MNA-SF). J Gerontol A Biol Sci Med Sci, 56, 2001, M366–M372, doi:10.1093/gerona/56.6.M366

S9. L.J.H. Rasmussen, J.E.V. Petersen, and J. Eugen-Olsen, Soluble urokinase plasminogen activator receptor (suPAR) as a biomarker of systemic chronic inflammation. Front Immunol, 12, 2021, doi:10.3389/fimmu.2021.780641.

S10. M. Roberts, M.D. Lindheimer, and J.M. Davison, Altered glomerular permselectivity to neutral dextrans and heteroporous membrane modeling in human pregnancy. *Am J Physiol*, **270**, 1996, F338–F343, doi:10.1152/ajprenal.1996.270.2.F338.

S11. R. Oberbauer, V. Nenov, C. Weidekamm, et al., Reduction in mean glomerular pore size coincides with the development of large shunt pores in patients with diabetic nephropathy. *Exp Nephrol*, **9**, 2001, 49–53, doi:10.1159/000020698.

S12. A.S. Levey, L.A. Stevens, C.H. Schmid, et al., A new equation to estimate glomerular filtration rate. *Ann Intern Med*, **150**, 2009, 604–612, doi:10.7326/0003-4819-150-9-200905050-00006.

S13. L.A. Inker, C.H. Schmid, H. Tighiouart, et al., Estimating glomerular filtration rate from serum creatinine and cystatin C. *New Eng J Med*, **367**, 2012, 20–29, doi:10.1056/NEJMoa1114248.

S14. L.A. Inker, N.D. Eneanya, J. Coresh, et al., New creatinine- and cystatin C-based equations to estimate GFR without race. *New Eng J Med*, **385**, 2021, 1737–1749, doi:10.1056/NEJMoa2102953.

S15. L.A. Inker, H. Tighiouart, M.O. Adingwupu, et al., CKD-EPI and EKFC GFR estimating equations: performance and other considerations for selecting equations for implementation in adults. *J Am Soc Nephrol*, **34**, 2023, 1953–1964, doi:10.1681/ASN.0000000000000227.

S16. H. Pottel, J. Björk, M. Courbebaisse, et al., Development and validation of a modified full age spectrum creatinine-based equation to estimate glomerular filtration rate: a cross-sectional analysis of pooled data. *Ann Intern Med*, **174**, 183–191, doi:10.7326/M20-4366.

S17. H. Pottel, J. Björk, A.D. Rule, et al., Cystatin C-based equation to estimate GFR without the inclusion of race and sex. *New Eng J Med*, **388**, 2023, 333–343, doi:10.1056/NEJMoa2203769.

STROBE Statement—Checklist of items that should be included in reports of ***cross-sectional studies***

	Item No	Recommendation	Page No
**Title and abstract**	1	(*a*) Indicate the study's design with a commonly used term in the title or the abstract	1
		(*b*) Provide in the abstract an informative and balanced summary of what was done and what was found	N/A
**Introduction**			
Background/rationale	2	Explain the scientific background and rationale for the investigation being reported	2–3
Objectives	3	State specific objectives, including any prespecified hypotheses	3
**Methods**			
Study design	4	Present key elements of study design early in the paper	3
Setting	5	Describe the setting, locations, and relevant dates, including periods of recruitment, exposure, follow-up, and data collection	3
Participants	6	(*a*) Give the eligibility criteria, and the sources and methods of selection of participants	Supplementary material
Variables	7	Clearly define all outcomes, exposures, predictors, potential confounders, and effect modifiers. Give diagnostic criteria, if applicable	3
Data sources/ measurement	8*	For each variable of interest, give sources of data and details of methods of assessment (measurement). Describe comparability of assessment methods if there is more than one group	Supplementary material
Bias	9	Describe any efforts to address potential sources of bias	N/A
Study size	10	Explain how the study size was arrived at	Supplementary material
Quantitative variables	11	Explain how quantitative variables were handled in the analyses. If applicable, describe which groupings were chosen and why	3
Statistical methods	12	(*a*) Describe all statistical methods, including those used to control for confounding	Supplementary material
		(*b*) Describe any methods used to examine subgroups and interactions	N/A
		(*c*) Explain how missing data were addressed	N/A
		(*d*) If applicable, describe analytical methods taking account of sampling strategy	Supplementary material
		(*e*) Describe any sensitivity analyses	3–4
**Results**			
Participants	13*	(a) Report numbers of individuals at each stage of study—eg numbers potentially eligible, examined for eligibility, confirmed eligible, included in the study, completing follow-up, and analysed	4
		(b) Give reasons for non-participation at each stage	Supplementary material

		(c) Consider use of a flow diagram	N/A
Descriptive data	14*	(a) Give characteristics of study participants (eg demographic, clinical, social) and information on exposures and potential confounders	4
		(b) Indicate number of participants with missing data for each variable of interest	N/A
Outcome data	15*	Report numbers of outcome events or summary measures	4
Main results	16	(*a*) Give unadjusted estimates and, if applicable, confounder-adjusted estimates and their precision (eg, 95% confidence interval). Make clear which confounders were adjusted for and why they were included	4
		(*b*) Report category boundaries when continuous variables were categorized	4
		(*c*) If relevant, consider translating estimates of relative risk into absolute risk for a meaningful time period	N/A
Other analyses	17	Report other analyses done—eg analyses of subgroups and interactions, and sensitivity analyses	4
**Discussion**			
Key results	18	Summarise key results with reference to study objectives	4–5
Limitations	19	Discuss limitations of the study, taking into account sources of potential bias or imprecision. Discuss both direction and magnitude of any potential bias	5
Interpretation	20	Give a cautious overall interpretation of results considering objectives, limitations, multiplicity of analyses, results from similar studies, and other relevant evidence	5–7
Generalisability	21	Discuss the generalisability (external validity) of the study results	5–7
**Other information**			
Funding	22	Give the source of funding and the role of the funders for the present study and, if applicable, for the original study on which the present article is based	8

*Give information separately for exposed and unexposed groups.

Note: An Explanation and Elaboration article discusses each checklist item and gives methodological background and published examples of transparent reporting. The STROBE checklist is best used in conjunction with this article (freely available on the Web sites of PLoS Medicine at http://www.plosmedicine.org/, Annals of Internal Medicine at http://www.annals.org/, and Epidemiology at http://www.epidem.com/). Information on the STROBE Initiative is available at www.strobe-statement.org.
